# Draft Genome Sequence of the Hydrogen- and Ethanol-Producing Anaerobic Alkalithermophilic Bacterium *Caloramator celer*

**DOI:** 10.1128/genomeA.00471-13

**Published:** 2013-07-18

**Authors:** Alessandro Ciranna, Antti Larjo, Anniina Kivistö, Ville Santala, Christophe Roos, Matti Karp

**Affiliations:** Department of Chemistry and Bioengineering, Tampere University of Technology, Tampere, Finlanda; Department of Signal Processing, Tampere University of Technology, Tampere, Finlandb; Department of Information and Computer Science, Aalto University, Helsinki, Finlandc; Euformatics Oy, Espoo, Finlandd

## Abstract

*Caloramator celer* strain JW/YL-NZ35 is a Gram-positive thermophilic, alkalitolerant, and strictly anaerobic bacterium capable of producing hydrogen and ethanol under extreme conditions. The draft genome sequence presented here will provide valuable information to further explore the physiology of this species and its potential for biofuel production.

## GENOME ANNOUNCEMENT

*Caloramator celer* strain JW/YL-NZ35, formerly known as *Thermobrachium celere* (equivalent to ATCC 700318 and DSM 8682) (1), is a Gram-positive thermophilic, alkalitolerant, and strictly anaerobic bacterium isolated from hot spring sediments (Ohinemutu, New Zealand), with an optimal growth temperature of 67°C, an optimal pH at 67°C of 8.2, and a doubling time reported to be as low as 10 min (2). During anaerobic fermentation, *C. celer* converts C_6_ sugars to H_2_, CO_2_, acetate, ethanol, and formate as major metabolites. Previous studies have shown that *C. celer* is able to produce hydrogen at high yields both in a naturally occurring microbial community (3) and in pure culture (4, 5). In addition, it can produce a significant amount of ethanol depending on the growth conditions. Recently, other members of the genus *Caloramator* were investigated for their biotechnological potential (6, 7), but only the genome of one species (*Caloramator australicus* RC3T) has been revealed (8). In order to evaluate the metabolism and the potential for biofuel production of the species *C. celer* and to expand the knowledge of the genus *Caloramator*, a draft genome sequence of strain *C. celer* JW/YL-NZ35 is presented.

The genome of *C. celer* JW/YL-NZ35 was sequenced with Illumina HiSeq 2000 to get paired-end reads from short (~250 bp) and long (~3 kbp) fragment libraries, as well as with 454 sequencing to get longer single-end reads. Assembly of the genome was performed with MIRA (9) followed by contig extension and scaffolding with SSPACE (10). The genome was annotated using the RAST server (11) and, when needed, manual assessment and curation of the automatic annotation were performed by BLAST analysis (12). The improved high-quality draft (13) genome assembly has a total size of 2,644,756 bp, organized in 56 scaffolds (>1 kb) (consisting of 162 contigs with an N_50_ of 128,968 bp), the longest being 1,976,539 bp. The G+C content of genome is 31.3%. On the basis of the annotation, the genome contains 2,381 protein-coding sequences (CDSs), including 151 RNAs.

Further genome analysis provides insights into the metabolic pathways leading to H_2_ and ethanol synthesis. Three operons coding for putative enzymes involved in the regeneration of NAD^+^ and oxidized ferredoxin through proton reduction with consequent H_2_ synthesis were identified: two putative heterotetrameric NADH-dependent [FeFe]-hydrogenases whose subunits have, respectively, from 47 to 67% and from 49 to 77% identity to genes TTE0890 to TTE0894 of *Thermoanaerobacter tengcongensis* (14), and one putative multimeric ferredoxin-dependent membrane-bound [NiFe]-hydrogenase whose subunits show from 30 to 54% identity to genes PF1423 to PF1436 of *Pyrococcus furiosus* (15). Alternatively, regeneration of NAD^+^ can be carried out by the conversion of acetyl-coenzyme A (CoA) to ethanol by two putative alcohol dehydrogenases. The *C. celer* draft genome sequence will allow a more systematic investigation of the potential of this organism for bioenergy applications and will expand the knowledge of the physiology of this genus.

### Nucleotide sequence accession numbers.

This whole-genome shotgun project has been deposited at DDBJ/EMBL/GenBank under the accession no. CAVN000000000. The version described in this paper is the first version, accession no. CAVN010000000.
